# The influence of insecure attachment on undergraduates’ jealousy: the mediating effect of self-differentiation

**DOI:** 10.3389/fpsyg.2023.1153866

**Published:** 2023-08-24

**Authors:** Min Deng, Endale Tadesse, Sabika Khalid, Weida Zhang, Junrong Song, Chunhai Gao

**Affiliations:** ^1^Humanities and Management School, Kunming Medical University, Kunming, Yunnan, China; ^2^College of Teacher Education, Zhejiang Normal University, Zhejiang, China; ^3^School of Education, Renmin University of China, Beijing, China; ^4^Computational Media and Arts, Hong Kong University of Science and Technology, Guangzhou, Guangdong, China; ^5^Faculty of Education, Shenzhen University, Shenzhen, China

**Keywords:** undergraduates, attachment, jealousy, mediating effect, differentiation

## Abstract

**Background:**

Jealousy is a complex emotion and can be healthy or pathological, depending on the intensity and the degree of control. Excessive jealousy was characterized by anxiety, anger, and alienation in the insecure attachment relationship.

**Objective:**

To explore how insecure attachment triggered this intense emotion, this study investigated the relationship between two insecure attachment dimensions and jealousy and explored the influence of self-differentiation on the relationship.

**Method:**

A total of 477 undergraduates participated in the study, and the Bringle self–report jealousy scale (BSJS), the relationship questionnaire (RQ), the intimate relationship experience questionnaire (ECR), and the revised edition of self–the differentiation questionnaire (DSR) were used.

**Result:**

The results showed that: (1) attachment anxiety had a significant positive predictive effect on jealousy, but attachment avoidance had no significant positive predictive effect; (2) self-differentiation partially mediated the relationship between attachment anxiety and jealousy, but it has no significant mediating effect between attachment avoidance and jealousy.

**Conclusion:**

The results suggest that attachment anxiety was correlated with jealousy because it strengthened the intensity of anxiety and anger toward their attachment figures and became out of control through a lower level of self-differentiation, which has important implications for clinical intervention.

## Introduction

Jealousy describes a range of negative, irrational, and excessive emotions. In the past two decades, envy has been understood by mainstream psychologists as a social construct or a pathological psychology created by capitalist society (Salovey and Rodin, 1988). According to the psychodynamic theory, jealousy originates from the fear and desire in an individual’s subconscious. Everyone has different anxiety and fear levels when separated from their attachment figure in childhood, so jealousy is universal and inevitable (Lerner, 1974). Bowlby (1969, 1982) argues that humans are born with an innate psychobiological system (attachment behavior system) that prompts people to seek closeness to significant others (attachment objects) when they need it. According to attachment theory, when individuals perceive threats in meaningful relationships, the attachment behavior system is activated, and individuals will seek closeness to regain a sense of security. When their desire is stopped by another confidential figure with more advantage, they will be lost in deep pain and jealousy. The attachment theory, which holds that jealousy is rooted in early attachment patterns and the quality of current relationships, has been supported by research. Rydell and Bringle (2007) and Marazziti et al. (2010) found that the jealousy level of insecure attachment individuals was significantly higher than that of secure attachment individuals. Marazziti et al. (2010) found that the level of jealousy of unsafe attachment individuals was significantly higher than that of safe attachment individuals, especially social jealousy related to interpersonal comparison and social competition, which was more prominent. Besides, Wenhui and Guangdong (2020) found that, for young men and women in romantic relationships, attachment anxiety and avoidance will affect their jealousy to varying degrees when they imagine their lovers touching their heterosexual friends. Thus emerging adults’ perceived insecurity in parental attachment may precede emerging adults’ emotions of jealousy (Kim et al., 2017).

Jealousy is an intense negative emotion for losing desire, inducing low intimacy, and not being tolerant of facing separation. According to the model of romantic jealousy, it is divided into reactive and preventive jealousy (White and Mullen, 1989). The former is healthy for keeping and maintaining a close relationship. However, the latter leads to pathological jealousy, aiming to capture a form of jealousy characterized by high emotional intensity and out of control by crazy anxiety, fear, and anger. A previous study indicated anxious attachment styles with jealousy induction, compared to avoidant attachment, because people with anxiety may be more prone to interpret situations as threatening to their relationships (Wegner et al., 2018). Highly jealous people try to keep excessive intimacy with their partners and avoid being alone or separated, quickly leading to insecure attachment.

Furthermore, the empirical evidence suggests a positive association between anxious attachment and reactive jealousy (Knobloch et al., 2001) and the exact positive correlation between avoidant attachment and suspicious jealousy (Güçlü et al., 2017). Based on these previous studies, we hypothesized that jealousy reaction would be related to attachment types and has a unique mechanism of action. Other significant obvious problems also show that individuals have difficulty dealing with the relationship between themselves and their partners, either by being too close or distant. To explain this complex relationship, we need to discuss the concept of self-differentiation. Differentiation of self (DOS) is the core concept of Bowen (1978) family system theory, which reflects the degree of emotional dependence and independence of individuals in the family. Specifically, DOS refers to the ability of individuals to distinguish between their emotions and reason and to experience intimacy and independence in relationships (Hazan and Shaver, 1987). From the point of view of individual development, as individuals grow, they need to be differentiated from symbiotic fusion with their mothers. Bowen (1978) believes there are always two primitive forces of individualization and intimacy in the individual’s heart, in which intimacy enables the individual to maintain a close connection with his family psychologically. Individualization promotes the separation of individuals from their families psychologically. The two are always in a state of confrontation, and the individual can develop well only by maintaining a dynamic balance (Bowen, 1976). Thus how to deal with this dynamic balance between intimacy and individualization is decided by the level of DOS.

Differentiation of self is an essential individual characteristic that helps people establish relationships with others by remaining independent (Pryor and Haber, 2009; Lampis et al., 2017; Lampis and Cataudella, 2019). As one is early attachment experience, the level of one’s DOS is relatively stable; highly DOS can maintain autonomy in intimate relationships and avoid emotional problems. In comparison, lowly DOS struggles to maintain intimate relationships and uses interpersonal alienation to relieve negative emotions.

Many researchers believe that DOS and attachment have an everyday theoretical basis; attachment theory proposes that the emotional bond between a child and her primary caregiver has necessary implications for psychological development, shaping interpersonal interaction models (Bowlby, 1969). Insecure attachment predicted a low level of DOS, and individuals with good self-differentiation were secure primary attachments. Only when an individual obtains a stable “safe base” in the secure attachment with the caregiver, he/she may not worry about losing the caregiver continuously and have enough energy to explore the outside world, complete the separation from the caregiver, and form a good DOS in the later psychological development (Bowen and Gillath, 2020).

Furthermore, anxious attachments are more likely to rely excessively on others to deal with negative emotions; Avoidance attachments with a tendency to perceive the self as positive and keep isolated with clothes others (Cassidy et al., 2010). They may represent different levels of DOS; Skowron and Dendy (2004) found that attachment style predicted aspects of DOS because anxious attachment was associated with higher levels of emotional reactivity, and avoidant attachment was associated with higher levels of emotional cutoff. Another study regarded that attachment styles affected the formation of DOS; they proposed that the basic level of DOS was developed throughout the attachment experience. Krems et al. (2021) further described two levels of differentiation-basic and functional differentiation. The former was primarily determined by early interaction attachment experience, but the latter was variable at different points in their lives, especially in specific emotional regulation, stress coping, and mental distresses. Therefore, we need to discuss the effect of different attachment styles on the level of DOS, and attachment may play an essential role in functional differentiation.

Not only is there a direct relationship between attachment and DOS, but also a direct relationship between DOS and jealousy. Telli and Yavuz Güler (2021) also reported a negative association between jealousy and DOS in Turkish participants. Although similar research is very few, there still imply some complex relationships among three factors. Emerging findings showed that higher levels of anxiety and avoidance attachment displayed lower levels of differentiation of self in adults with anxiety disorders (Xue et al., 2018). Another study explored the relationship between self-differentiation and jealousy and found that anxious attachment is entirely mediated (Haibo and Qingzhu, 2015). It is suggested that the level of DOS plays a more significant role in the relationship between attachment anxiety and jealousy.

In order to explore whether insecure attachment can negatively predict DOS and positively predict jealousy and distinguish the different effects of DOS on attachment anxiety and avoidance, we hypothesize that DOS plays a mediating role between attachment anxiety, attachment avoidance, and jealousy, and the mediating role is more significant in attachment anxiety than in attachment avoidance.

## Method

### Participants

G*Power3.1.9.2 was used for preliminary analysis of the required sample size. The effect size was moderate effect (*F* = 0.25), the significance level was α =0.05, the power was 0.80, and the total sample size was at least 34. Participants were emerging adults who were university students at the undergraduate level in China. All of them had been in a relationship for more than a year and had been excluded from the group that was not in a relationship. Volunteers participated in the study by answering the questionnaires in a spot on the university campus. Four hundred eighty-five questionnaires were distributed, 477 valid questionnaires were recovered, and the recovery rate was 98.35. Of these, 262 were boys, and 215 were girls, aged between 17 ~ and 23, with an average age (of 20.37 ± 1.09) years.

### Measures

#### Bringle self-report jealousy scale

We used the Self-Report Jealousy Scale (SRJS) to measure two aspects of jealousy in adult romantic relationships. This scale was first developed by Bringle et al. (1979). The SRJS has been validated in many studies (Bringle et al., 1983; Bringle, 1991). A total of 20 items, including love jealousy and social jealousy dimensions. And social jealousy can be subdivided into social competition jealousy and interpersonal jealousy. A score of 1 (somewhat jealous) ~ 5 (very jealous) was used. The sum of the scores of each item represents the level of jealousy of the individual. The higher the score, the stronger the jealousy. The internal consistency coefficients of the total scale and the three dimensions in our study were love jealousy (7 items, *α* = 0.78), social jealousy (5 items, *α* = 0.60), interpersonal jealousy (8 items, *α* = 0.69), and total jealousy (20 items, *α* = 0.82). The confirmatory factor analysis of the model was carried out with AMOS, and the factor model was within the acceptable range (X^2^/df = 4.615 < 5, RMSEA = 0.086 < 0.09, CFI = 0.750 > 0.7, TLI =0.716 > 0.7).

#### Experience in close relationship questionnaire

We used the Experiences in Close Relationships Scale (ECRS) to measure two adult romantic attachment style aspects. This scale was developed by Brennan et al. (1998) and later validated by Mikulincer and Shaver (2007). Li and Kato (2006) revised the Chinese version of the ECR scale (ECR-R), including the attachment avoidance subscale and attachment anxiety subscale, of which Cronbach’s coefficients were 0.82 (attachment avoidance) and 0.77 (attachment anxiety), and the retest reliability were 0.7 L (attachment avoidance) and 0.72 (attachment anxiety), respectively. This study uses ECR-R to measure one’s attachment dimension to adapt Chinese context. Measure the adult attachment style of emerging adults. There are two dimensions of attachment anxiety and avoidance, each containing 18 items. Adopt 1 (completely inconsistent) ~ 7(completely consistent) score. The internal consistency reliability of attachment avoidance and anxiety dimensions were 0.71 (attachment avoidance) and 0.78 (attachment anxiety), respectively. Cronbach’s α coefficient and combined reliability (CR) were used to test the reliability of the questionnaire. Both α coefficient and CR were greater than 0.7, indicating the scale’s reliability. AOMS carried out the confirmatory factor analysis of the model, and the factor model was within the acceptable range (X^2^/df = 3.916 < 5, RMSEA = 0.078 < 0.08, CFI = 0.612, TLI = 0.565).

#### Revised self–differentiation questionnaire

The self-differentiation scale is, revised by Chinese scholars Yuhui and Guiping (2010), mainly used to evaluate the degree of self-differentiation in adults. This study uses a revised version conducted by domestic scholars. The revised scale has good reliability and validity and meets the requirements of psychological measurement. The degree of self–differentiation of emerging adults was measured, including four dimensions: emotional reactivity (ER), self–position (IP), emotional disconnection (EC), and fusion (FO) with others, with a total of 27 items. Adopt 1 (completely inconsistent) ~ 6 (entirely consistent) score; the higher the score, the better the ability of self–differentiation. The internal consistency coefficients of the total scale and the four dimensions in our study were fusion with other (10 items, 0.79), self-position (5 items, *α* = 0.71), emotional breakdown (6 items, *α* = 0.75), emotional reactivity (6 items, *α* = 0.72) and total self-differentiation (25 items, *α* = 0.82). AOMS carried out the confirmatory factor analysis of the model, and the factor model was within the acceptable range (X^2^/df = 3.594 < 5, RMSEA = 0.073 < 0.08, CFI = 0.766 > 0.7, TLI =0.742 > 0.7).

### Procedure

The data were collected using random cluster sampling at Kunming Medical University. All participants who read an informed consent document before the survey could stop participating at any time without penalty and could get a reward of 5-RMB after finishing the measures.

### Quantitative analyses

SPSS Version 22.0 is used to analyze the data. Because all the variables were self–reported, Harman’s single factor was used to test whether there was a standard method deviation. Exploratory factor analysis indicated that the questionnaire data met the prerequisite requirements of factor analysis with the value of KMO = 0.832, more significant than 0.7, and Bartlett’s spherical test value reached significant (*p* < 0.001). The results show that the percentage of variance interpretation of the first common factor is 12.94, less than the critical value of 40%, indicating no apparent standard method deviation. Metrology data accord with a normal distribution (M ± SD). Correlation analysis adopts Pearson correlation analysis, and regression analysis adopts multiple linear regression; the difference reached statistical significance (*p* < 0.05).

## Results

### Descriptive statistics

The descriptive statistics are shown in Table 1. The participants scored low in jealousy and medium self–determination, attachment anxiety, and attachment avoidance. All the variables showed no difference in sex.

**Table 1:** Test for sex differences between scores of an emerging adults with different study projects.

Project	General sample (n = 477)	Sex		*t*
		Male (*n* = 262)	Female (*n* = 215)	
Attachment anxiety	3.60 ± 0.81	3.63 ± 0.85	3.58 ± 0.77	−0.82
Attachment avoidance	3.47 ± 0.80	3.37 ± 0.78	3.58 ± 0.80	−3.43
Love jealousy	3.07 ± 0.77	3.01 ± 0.77	3.12 ± 0.76	−1.95
Interpersonal jealousy	2.37 ± 0.75	2.30 ± 0.73	2.44 ± 0.76	−2.51
Social competition jealousy	2.78 ± 0.72	2.70 ± 0.76	2.86 ± 0.67	−2.75
Total jealousy	2.78 ± 0.61	2.71 ± 0.62	2.85 ± 0.59	−3.05
Emotional Reactivity	3.41 ± 081	3.51 ± 0.75	3.30 ± 0.86	−1.37
Self–position	4.07 ± 0.84	4.10 ± 0.92	4.03 ± 0.75	−2.01
Emotional breakdown	3.88 ± 0.85	3.86 ± 0.88	3.90 ± 0.82	1.24
Integration with others	3.49 ± 0.76	3.53 ± 0.78	3.44 ± 0.73	−0.42
Total self–differentiation	3.71 ± 0.53	3.75 ± 0.54	3.66 ± 0.51	−1.39

Correlation analysis of attachment dimension, jealousy experience, and self–differentiation.

The scores of self–differentiation were significantly negatively correlated with attachment anxiety, attachment avoidance, love jealousy, interpersonal jealousy, social and competitive jealousy, and total score of jealousy experience but positively correlated with emotional reactivity, self–position, emotional disconnection, and integration dimension with others. The relationship between the three dimensions of jealousy experience and attachment is more complex. Love jealousy and attachment anxiety are significantly positively correlated but negatively correlated with attachment avoidance, and interpersonal jealousy is negatively correlated with unsafe attachment. Jealousy was only positively correlated with attachment anxiety in the social competition dimension but failed to reach a significant level of attachment avoidance (Table 2).

**Table 2:** The correlation analysis of attachment dimensions, jealousy experience, and DOS(*r*).

Variable	1	2	3	4	5	6	7	8	9	10	11
1. Attachment anxiety	1.00										
2. Attachment avoidance	0.49^**^	1.00									
3. Love jealousy	0.26^**^	0.17^**^	1.00								
4. Interpersonal jealousy	0.21^**^	0.10^*^	0.25^**^								
5. Social competition envy	0.24^**^	0.21^**^	0.57^**^	0.48^**^							
6. Total jealousy	0.30^**^	0.19^**^	0.76^**^	0.78^**^	0.81^**^						
7. Emotional reactivity	0.22^**^	0.16^**^	0.21^**^	0.08	0.24^**^	0.21^**^					
8. Self–position	0.19^**^	−0.06	−0.20^**^	0.26^**^	0.03	0.05	−0.05				
9. Emotional breakdown	0.26^**^	0.14^**^	−0.03	0.31^**^	0.13^**^	0.19^**^	0.32^**^	0.20^**^			
10. Integration with others	0.40^**^	0.31^**^	−0.18^**^	0.21^**^	0.28^**^	0.28^**^	0.48^**^	0.08^**^	0.56^**^		
11. Total self–differentiation	0.44^**^	0.25^**^	0.14^**^	0.37^**^	0.32^**^	0.36^**^	0.62^**^	0.38^**^	0.74^**^	0.85^**^	1.00

^*^p < 0.05, ^**^p < 0.01, *n* = 477.

### The mediating effects of DOS between attachment anxiety, avoidance, and jealousy

First, all the variables were standardized, and then the moderated mediating effect analysis was conducted in two steps under the condition of controlling sex, age, and love experience. All the analysis processes were carried out by SPSS macro program PROCESS. The percentile Bootstrap method with deviation correction was used for the test, repeated sampling 5,000 times, and 95% confidence intervals were calculated. Specific results are shown in Table 3.

**Table 3:** Regression analysis between attachment and jealousy: the mediating role of DOS.

Dependent variable	DOS (Step 1)		DOS (Step 1)		Jealousy (Step 1)		DOS (Step 2)	
Independent variable	*β*	*t*	*β*	*t*	*β*	*t*	*β*	*t*
Age	−0.01	−0.15	−0.04	−0.92	0.07	1.64	0.06	1.30
Sex	0.01	0.10	0.02	0.03	0.12	2.66	0.12	2.87
Love experience	−0.02	−0.52	−0.05	−1.04	−0.02	−0.43	−0.02	−0.37
Attachment anxiety	0.26	6.97			0.26	6.97^***^	0.16	3.92^***^
Attachment avoidance			0.16	3.92	0.22	4.30^***^	0.13	2.53^***^
DOS							0.20	4.40^***^
*R^2^*	0.10		0.16		0.04		0.14	
*F*	48.61^***^		34.29^***^		18.48^***^		55.22^***^	

^***^p < 0.001.

**Figure 1:**
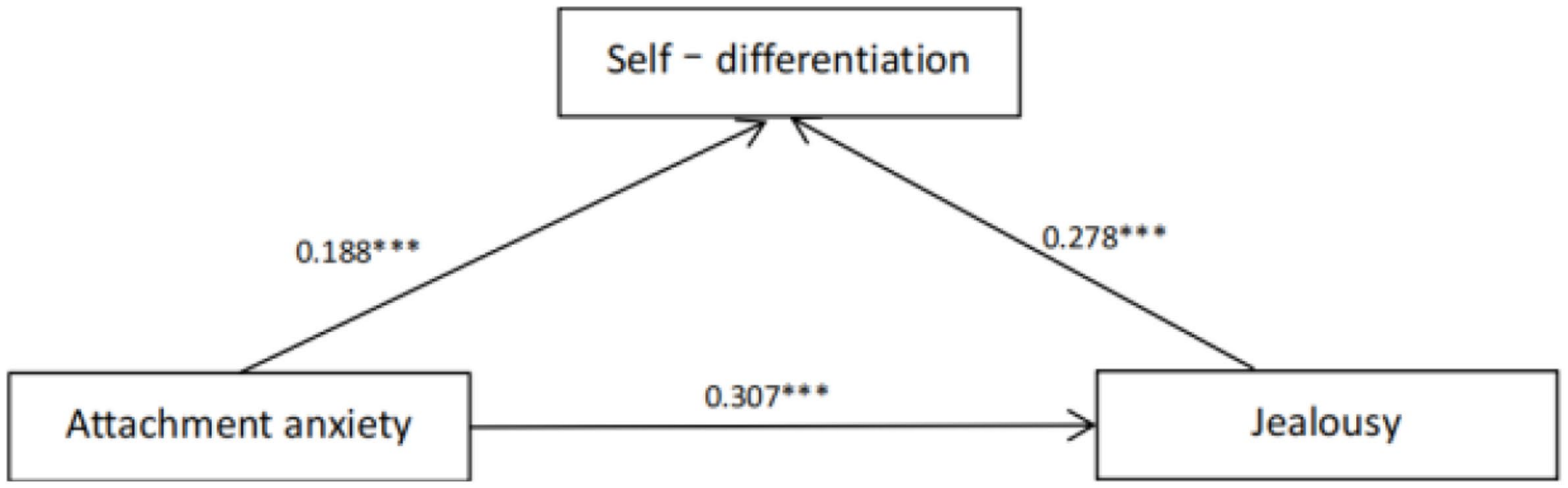
The effect of attachment anxiety on jealousy is mediated by self-differentiation. ***p < 0.001.

**Figure 2:**
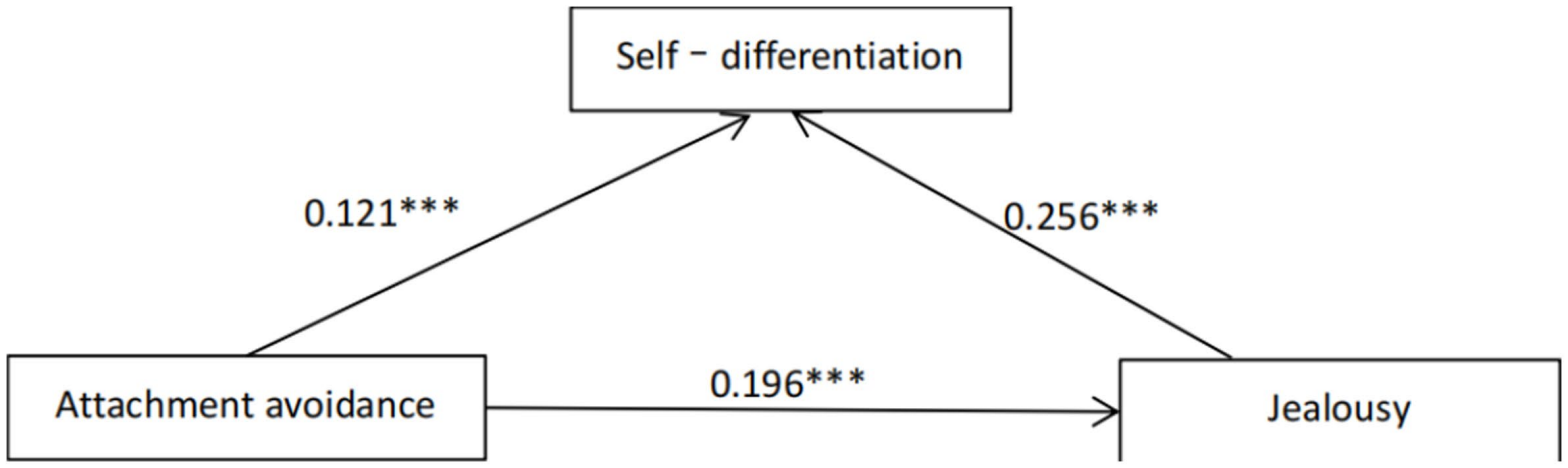
The effect of attachment avoidance on jealousy is mediated by self-differentiation. ***p < 0.001.

In order to investigate the predictive relationship and intermediary effect of attachment anxiety and attachment avoidance between emerging adults’ jealousy experience and DOS, under the influence of controlling sex, age, and love experience, jealousy experience was taken as the dependent variable, and attachment anxiety/avoidance was divided into independent variables. Stepwise regression analysis was carried out. The results showed that the scores of attachment anxiety, attachment avoidance, and self–differentiation were all negatively correlated, and the independent variables explained 25 percent of the variation degree of dependent variables (Table 3 and Figures 1, 2).

From the regression result, the next step is to test the simple mediation model. Among moderated mediation models, the simple mediation model is the benchmark model, so it is tested first (Hayes, 2013; Wen and Ye, 2014). First, model 4 was selected to examine the mediating role of self-differentiation between attachment anxiety/attachment avoidance and jealousy. Regression analysis showed that controlling for sex, age, and love experience, attachment anxiety had a significant positive predictive effect on jealousy (β*attachment anxiet*y = 0.26, *t* = 6.97, *p* < 0.001); Attachment avoidance had a significant positive predictive effect on jealousy (β*attachment anxiety* = 0.22, *t* = 4.30, *p* < 0.001); When self-differentiation was included in the regression equation, the predictive effect of attachment anxiety on jealousy was still significant (β*attachment anxiety* = 0.16, *t* = 3.92, *p* < 0.001); Attachment avoidance was significant in predicting jealousy (β*attachment avoidance* = 0.13, *t* = 2.53, *p* < 0.001), self-differentiation positively predicted jealousy (β*self-differentiation* = 0.20, *t* = 4.40, *p* < 0.001). Boot SEattachment anxiety =0.02, 95% confidence interval was [0.02, 11], indicating that self-differentiation partially mediated the relationship between attachment anxiety and jealousy. While Boot SEattachment avoidance =0.02, 95% confidence interval is [0.03,0.14], attachment avoidance has also a significant predictive effect on jealousy, indicating that self-differentiation has a significant mediating effect between attachment avoidance and jealousy.

## Discussion

Based on these two conditions of insecure attachment, we constructed different mediation models, and the results showed that attachment anxiety was significantly correlated with the total score of jealousy and jealousy dimensions. This Result is consistent with Costa et al. (2015) and Richter et al. (2022). Diotaiuit and colleagues conducted a validation study of the Italian version of the multidimensional jealousy scale, which showed that anxiety and jealousy were positively correlated and supported this study (Diotaiuti et al., 2022). According to attachment theory, individuals with attachment anxiety fear abandonment. According to the study by Cassidy et al. (2010), anxious attachment types are willing to rely on others to solve negative emotions, so they crave intimacy and fear loneliness in intimate relationships. So you are more likely to be threatened and more likely to feel jealous under normal stimuli. In terms of emotional response, existing studies have found that attachment style can predict the level of self-differentiation, and anxious attachment is associated with a higher level of emotional response, which also verifies the significant negative correlation between anxious attachment and self-differentiation level in this paper (Skowron and Dendy, 2004). Based on the study of Skowron and Dendy (2004), we considered that anxious attachment is associated with a higher level of emotional response, so the level of self-differentiation is low, and attachment-anxious individuals tend to deal with problems emotionally in an intimate relationship. In this process, attachment-anxious individuals are more inclined to maintain an excessively close relationship with others and lose their independence. So this mediation model shows that anxiety further influences jealousy by affecting the level of self-differentiation.

Studies show that attachment anxiety and avoidance style was positively correlated with cognitive, emotional and behavioral types of jealousy. The same study also found that the insecure attachment style was positively correlated with cognitive jealousy (Attridge, 2013). Jealousy is usually related to the individual’s irrational cognitive factors, often seeing many factors as negative or bad. Jealousy is often associated with low self-esteem, low confidence, low trust and other factors, indicating negative perceptions and unreasonable beliefs about the self, which is consistent with the internal working model of attachment anxiety. Although the self-recognition of attachment avoidance is positive, it also shows negative cognitive and emotional experience under high cognitive load. Similar results were also shown in this study. Both attachment anxiety and attachment avoidance were positively correlated with jealousy, and attachment anxiety was more closely correlated with jealousy. The results of this study suggest that attachment avoidance also has less jealousy. However, unlike secure attachment, attachment avoidance does not involve experiencing more intimate relationships, but maintaining relatively independent interpersonal distancing in intimate relationships, tending to keep a distance from others. Although jealousy experience is lower, the level of self-differentiation is also lower.

According to the different roles of attachment anxiety and attachment avoidance in self-differentiation and jealousy, this study further verified the research idea, constructed two mediation models of insecure attachment on jealousy, and obtained the different paths of two types of insecure attachment through self-differentiation. The mediating effect of attachment anxiety, self-differentiation, and jealousy is verified, and the specific application of self-differentiation in attachment theory is revealed. This is the combination of self-differentiation theory and attachment theory. It is also rare in attachment research to separate the two types of insecure attachment to explore the influence of self-differentiation on jealousy, expanding the theoretical research scope of self-differentiation.

### Limitations

This study mainly focuses on the intermediary role of jealousy experience in the relationship between attachment anxiety/avoidance and jealousy. It only considers the university students as the research object, which may lead to biased results. China is a collectivist cohort and thus may emphasize one’s intimate relationship. As a previous result, this was not sufficiently grounded; there is still a need for more research evidence to support the difference in self-differentiation development between collectivism and individualism and further discuss the change of these two mediating effects under different cultural contexts. In addition, given the complex interrelationship between unsafe attachment and other factors such as social factors, family education style, and personal experience, the interaction between multiple factors needs to be further discussed in the future, and the active intervention of emerging adults based on attachment should also be considered.

### Practical implications

The results may be helpful to undergraduate work practice because of providing counselors with a more sophisticated understanding of the interplay between jealousy, attachment, and self-differentiation. Counselors should focus on increasing intimacy in undergraduates’ relationships (i.e., familial, romantic, or friendship), increasing awareness and regulation of emotions, and separating emotions from thoughts (Skowron, 2000). This study may present a clearer picture of the influence of attachment. Because self-differentiation exists in all people to some degree and attachment occurs in and influences people’s emotionally essential relationships, the understanding of intimacy provided by the study’s results could potentially be applied to several kinds of relationships, including, in some ways, the therapeutic relationship (Neuenschwander, 2010).

## Data availability statement

The raw data supporting the conclusions of this article will be made available by the authors, without undue reservation.

## Ethics statement

The studies involving human participants were reviewed and approved by Kunming Medical University. The patients/participants provided their written informed consent to participate in this study.

## Author contributions

MD, CG, and SK participated in the conceptualization, methodology, writing, and editing. ET, WZ, and JS participated in the supervision, revision, and project administration. All authors have read and agreed to the published version of the manuscript.

## Funding

This project was supported by the Annual 2022 Joint Project of Basic Research in local universities (part) in Yunnan province (202101BA070001-156).

## Conflict of interest

The authors declare that the research was conducted in the absence of any commercial or financial relationships that could be construed as a potential conflict of interest.

## Publisher’s note

All claims expressed in this article are solely those of the authors and do not necessarily represent those of their affiliated organizations, or those of the publisher, the editors and the reviewers. Any product that may be evaluated in this article, or claim that may be made by its manufacturer, is not guaranteed or endorsed by the publisher.
